# Olfactory nerve: from ugly duckling to swan

**DOI:** 10.1590/0004-282X-ANP-2020-0529

**Published:** 2022-02-28

**Authors:** Sofia Mermelstein, Victor Evangelista Rodrigues Pereira, Paulo de Lima Serrano, Rachel Alencar de Castro Araújo Pastor, Abelardo Queiroz Campos Araujo

**Affiliations:** 1 Universidade Estadual do Rio de Janeiro, Hospital Universitário Pedro Ernesto, Departamento de Neurologia, Rio de Janeiro RJ, Brazil. Universidade Estadual do Rio de Janeiro Hospital Universitário Pedro Ernesto Departamento de Neurologia Rio de Janeiro RJ Brazil; 2 Universidade Federal do Rio de Janeiro, Hospital Universitário Clementino Fraga Filho, Departamento de Neurologia, Rio de Janeiro RJ, Brazil. Universidade Federal do Rio de Janeiro Hospital Universitário Clementino Fraga Filho Departamento de Neurologia Rio de Janeiro RJ Brazil; 3 Universidade Federal do Rio de Janeiro, Instituto de Neurologia Deolindo Couto, Departamento de Neurologia, Rio de Janeiro RJ, Brazil. Universidade Federal do Rio de Janeiro nstituto de Neurologia Deolindo Couto Departamento de Neurologia Rio de Janeiro RJ Brazil; 4 Fundação Oswaldo Cruz, Instituto Nacional de Infectologia Evandro Chagas, Rio de Janeiro RJ, Brazil. Fundação Oswaldo Cruz Instituto Nacional de Infectologia Evandro Chagas Rio de Janeiro RJ Brazil

**Keywords:** Clinical Neurology, Smell, Infectious Disease, Olfactory Nerve, Neurologia Clínica, Olfato, Doenças Infecciosas, Nervo Olfatório

## Abstract

**Background:**

The olfactory nerve has never been the shining star of neurological examination. Quite the contrary, examining the first cranial nerve is often an overlooked step. As cases of anosmia secondary to COVID-19 infection continue to rise, the 2020 pandemic has shed new light on this much-forgotten nerve, its value as an aid to diagnosis of several diseases and its central role in our daily lives.

**Objective:**

We aimed to emphasize how essential and simple clinical examination of the olfactory system can be by highlighting practical techniques and clinical tips for its assessment. We also share pearls and pitfalls in localization and differential diagnosis, which may prove valuable to busy clinicians.

**Methods:**

A broad review of the literature was conducted by searching PubMed, Cochrane and Google Scholar for articles and books containing topics regarding examination of the olfactory nerve and its anatomy, physiology and pathology. No particular inclusion or exclusion criteria were used.

**Results:**

Forty different works were found, between books and articles, from which 20 were selected after careful analysis.

**Conclusions:**

Despite the tragedy and adversity that followed the COVID-19 pandemic, its legacy has taught us a crystal-clear lesson: olfaction should no longer be neglected in clinical practice.

## RESUMO

**Antecedentes:**

O nervo olfatório nunca foi a estrela do exame neurológico. Pelo contrário, o exame desse nervo craniano é um passo frequentemente ignorado. No entanto, o aumento exponencial de casos de anosmia secundária a COVID-19 o colocou sob os holofotes, tanto em relação á sua função para o ser humano em sociedade, como seu papel no auxílio do diagnóstico de diversas patologias.

**Objetivos:**

Enfatizar quão importante é examinar o nervo olfatório e compreender as desordens do seu sistema. Ressaltamos pérolas clínicas e erros comuns no exame deste nervo, além dicas que possam auxiliar no diagnóstico de uma série de doenças neurológicas e sistêmicas.

**Métodos:**

Uma ampla revisão da literatura foi conduzida por meio de busca no PubMed, Cochrane e Google Acadêmico por artigos e livros relacionados aos tópicos do exame físico, fisiologia, anatomia e patologia do nervo olfatório. Não foram utilizados critérios específicos de inclusão ou exclusão.

**Resultados:**

Foram encontrados 40 artigos itens relacionados na língua inglesa, dentre os quais livros e artigos, tendo sido analisados e selecionados um a um até o total de 20 referências.

**Conclusões:**

Apesar da tragédia e adversidade trazidas pela pandemia de COVID-19, uma lição clara permanece: o olfato não deve mais ser negligenciado na prática clínica.

## INTRODUCTION

Intrinsically different, as primitive as it is sophisticated, the olfactory system does not play by the book. One example of its uniqueness is the fact that olfaction is the only human sense not to be firstly processed by the thalamus before reaching the cerebral cortex. However, olfaction does share similarities with other parts and properties of the human body, as it may benignly lose its function with age (presbyosmia) or reveal life-threatening organic diseases, intracranial lesions, systemic disorders or neurodegenerative conditions^1^.

Human olfaction has played such an admirable role during evolution that 2% of the entire human genome is concerned solely with expression of unique and distinct olfactory receptors^2^. Some vertebrate animals with highly developed olfactory systems may even have an “olfactory area” in their brains, occupying a proportionally identical space to that of the visual system.

Throughout history, the task of uncovering the mysteries of olfaction was not lost to humanity. Hippocrates’ account was the earliest basic anatomical description of the nose, followed by Leonardo da Vinci’s (1452-1519) description of the nasal conchae and sinuses^3^. Joseph-Hippolyte Cloquet (1787-1840) was the first physician to suggest, in his doctoral thesis “*On odours, the sense of olfaction and the olfactory organs”* (1815), the molecular nature of the odorous substrate, stating that*: ‘Olfaction can be seen at every turn of the labyrinth’*. More recently, in 1862, Max Schultze (1825-1874), an anatomy professor in Bonn, uncovered the olfactory sensory cell^4^, and in 1991, Richard Axel and Linda Buck’s work shed light on the hundreds of genes responsible for the odorant sensors in the olfactory neurons of the nose, for which they won the Nobel Prize in 2004^5^.

Despite several breakthroughs in the science of olfactory chemical stimuli, the mysteries of olfaction are probably still beyond the scope of modern scientific understanding. Smell plays an irreplaceable part in human communication, interaction, memory and emotion that is yet to be explained. This should not come as a surprise, considering the shared anatomical pathways between the olfactory and limbic systems within the brain. 

Smell evokes memories as complex as living sensations of experiences from the past. This connection intrigued and mesmerized the author of one of the greatest novels ever written: *“In Search of Lost time”*, Marcel Proust (1871-1922), who wrote about a vivid involuntary autobiographical memory triggered by the smell and taste that resulted from dipping a madeleine into a cup of tea, which henceforth became known as the “petit madeleine phenomenon”^6^. He beautifully wrote: *“But when from a long-distant past nothing subsists, after the people are dead, after the things are broken and scattered, taste and smell alone, more fragile but more enduring, more immaterial, more persistent, more faithful, remain poised a long time, like souls, remembering, waiting, hoping, amid the ruins of all the rest; and bear unflinchingly, in the tiny and almost impalpable drop of their essence, the vast structure of recollection”*^7^.

The importance of olfaction is, therefore, unquestionable. It is as central to many activities of daily living as it is to clinical practice. For example, smell can provide crucial sensory information in life-threatening situations like detecting the presence of smoke or toxic gas, or even interpreting taste stimuli in poisonous or spoiled food^8^. 

Bedside assessment of the olfactory system remains incredibly simple and straightforward, dispensing the need for expensive ancillary examinations. Despite the inherent importance and ease of examination of the olfactory nerve, it undoubtedly remains the most neglected cranial nerve in neurological practice^9^. As modern physicians looking back in history, we may even hypothesize about extent to which this omission might have impacted Neurology. Could it have been the explanation behind such curiosities as the fact that motor manifestations of Parkinson’s disease were described before prodromal non-motor manifestations like olfactory loss^10^? 

Our main purpose in writing this review was also a plea to all neurologists: we aimed to disseminate the practice of quick and objective examination of olfaction. We thus hoped to shed light on what the most important facts to be gathered from the clinical history might be, the pearls and pitfalls to avoid in examinations, some tips concerning localization and the clinical reasoning behind the myriad of differential diagnoses that may be causing olfactory deficits. In this manner, we expect to encourage further clinical study of the first cranial nerve, which could both facilitate discoveries in this field and improve our own diagnostic accuracy as practicing neurologists. 

## OLFACTORY NERVE EVALUATION: HOW TO APPROACH IT?

### What to ask? The clinical history

*Opening and chief complaints*

One of the main agreed-upon rules of general Neurology is that the patient’s history should be the cornerstone, providing a guide to all subsequent clinical reasoning. This also applies to olfactory evaluation. However, although patients with olfactory deficits often present with complaints of either loss of or diminished sense of smell (or even altered discrimination of different odors), this is not always the case. The assumption that normal smelling is so intuitive that the perception of any slight dysfunction must be readily evident to anyone is false and should work as a turning point in the mindset of the clinician taking the patient’s history. It is essential to remember that only 40% of patients with olfactory dysfunction will ever even notice it and, therefore, refer to it. Consequently, the absence or presence of spontaneous clinical complaints regarding loss of olfaction does nothing to absolutely exclude or confirm the existence of smell dysfunction. 

Another (often forgotten) caveat is that most patients who do complain of anosmia actually suffer from bilateral olfactory lesions. This is because unilateral lesions most often cause subclinical deficits, since contralateral olfactory function is preserved and can partially compensate for unilateral loss. This information is paramount, especially if a central nervous system mass lesion is suspected, which usually only injures the ipsilateral olfactory nerve. Consequently, unilateral deficits will probably only be detected through neurological examination, not from the patient’s history^2^.

A lack of spontaneous olfactory complaints does not mean that the clinical history is in any way dispensable. Faced with any evidence of memory loss, rigidity, tremor or parkinsonism (symptoms and signs that could be accompanied by subclinical olfactory deficits), detailed questioning and thorough examination of smell function is always mandatory^11^.

It is sometimes important to understand and evaluate disorders of smell and taste as a whole. Many patients who seek medical attention complaining of “loss of taste" or of “food tasting bland” actually have an underlying olfactory dysfunction. This results from the direct influence that our ability to distinguish between different types of odors has on our sense of taste. 

So, anosmia will actually often be presented to clinicians as a complaint of ageusia (loss of taste, not smell). More often than not, an organic loss of smell, even without true hypogeusia, is indeed associated with a subjective feeling that the food has become tasteless. However strong this sensation may be, it should not impair a patient's ability to distinguish between the five most elementary tastes: savory, sweet, sour, bitter and umami. If this distinction is lost, the patient probably does suffer from true ageusia or hypogeusia. Therefore, it is essential to determine which systems are affected: is the patient presenting with smell loss alone? Is it taste loss alone? Is it both, or neither? This information is useful not only with regard to differential diagnosis, but also when dealing with functional or simulating patients, as they may complain of complete loss of smell but with a completely unaltered sense of gustation, which, as we have stated, is very uncommon^8^. 

*Pearls in the history: etiology and topography*

Also in agreement with neurological clinical reasoning, olfactory dysfunction can result from either a central or a peripheral cause. One should always bear in mind when thinking about differential diagnosis that the second of these is more frequently observed than the first. Akin to hearing loss, a peripheral olfactory loss might be due to a neurosensorial or conductive problem. In conductive disorders, resulting most frequently from hypertrophy of the nasal mucosa, the pathway of odorants to the receptors in the olfactory cleft is obstructed, while in neurosensorial causes, it is the receptors themselves, not the pathway, that are damaged^12^.

Some elements in the patient’s history, such as epistaxis, nasal discharge or obstruction, are suggestive of conductive causes. Periods of worsening and improvement (fluctuation) of smell function during the course of a day, with remissions during physical activity, after taking a warm shower or through improvement with corticosteroid therapy (given that this reduces edema of the submucosal tissue, thus improving nasal congestion), also point to an obstructed pathway. 

One exception to the “fluctuating symptoms rule” in conductive causes is hyposmia secondary to viral upper respiratory tract infections (URTI). Albeit a “nasal cause”, hyposmia is usually continuous and is probably explained by concomitant neurosensorial dysfunction. Another clue to making the diagnosis is seasonality, since cases of smell loss due to URTI are more common during “flu seasons”. Moreover, as these patients start to recover, they characteristically present distortion of the perception of smell (dysosmia) or a sensation of smelling “ghost” odors that are not there (phantosmia)^13^. Likewise, we speculate that olfactory dysfunction secondary to SARS-CoV-2 behaves similarly.

Conversely, strictly neurosensorial etiologies often present with continuous and progressive symptoms, while complaints of nasal obstructions will mostly be absent. With regard to the time taken from onset of symptoms to progression to complete anosmia, neurosensorial peripheral disorders will usually evolve more rapidly and acutely than will dysfunction secondary to a central etiology, which often has a slower clinical course. In order to explain the reason behind this phenomenon, it is once again helpful to borrow from the example set by central hearing loss. Inside the central nervous system, bilateral connections form a dense, redundant network that compensates for any dysfunction until severe or widespread lesions are found^2^.

Particular details in the patient’s history can provide interesting clinical pearls that may help steer clinicians in the direction of particular etiologies. For instance, when someone reports that they do not remember ever having smelled anything in their entire life, this most likely means this person has suffered from olfactory dysfunction since birth (e.g., through congenital causes). One example is Kallmann syndrome^14^. 

Another syndrome worth mentioning is that of patients who complain of unilateral anosmia ipsilateral to the loss of multiple senses. They usually present with a very particular set of symptoms: anosmia on the same side as monocular visual loss, hearing loss and hemianesthesia. This should point to the diagnosis of a nonorganic olfactory dysfunction^1^.

A thorough exploration of the past medical history is needed, concerning events such as URTI, head injury, nasal surgeries, chemotherapy and radiotherapy, as well as exposure to conventional drugs and toxic agents. These questions may hold the key to the diagnosis. The hazardous effects of organic solvents, heavy metals, chemotherapy, cocaine, corticosteroids, methotrexate, aminoglycosides, tetracyclines and opioids are potentially harmful to the olfactory epithelium. Similarly, many conventional drugs commonly used in neurological practice can result in reversible hyposmia (e.g., valproic acid, levodopa or phenytoin)^12^.

*Non-hyposmic complaints*

It is important to keep in mind that not all olfactory complaints are identical, since they are not always related to diminished/loss of function (quantitative symptoms). Some patients present with “qualitative symptoms” such as olfaction distortion (e.g., dysosmia, as previously mentioned), difficulty in differentiating smells (as is the case in olfactory agnosia; see Tables 1 and 2), or even olfactory hallucinations. Whatever the nature of a particular olfactory complaint might be (qualitative or quantitative), reviewing other relevant signs and symptoms related to systemic or neurological diseases is imperative^2^.

**Table 1.** Glossary of olfactory terms: quantitative disorders.

Loss or reduction of smelling capacity	Increase of smelling capacity
Hyposmia: increased threshold for detecting odors.	Hyperosmia: diminished threshold for detecting odors.
Anosmia: complete loss of odor perception.	
Microsmia: decreased olfactory spectrum (differentiation of distinct odors).	
e.g. allergic rhinitis, neuronal damage after upper respiratory tract infection and olfactory groove meningioma.	e.g. migraine, anxiety and pregnancy.

**Table 2.** Glossary of olfactory terms: qualitative disorders.

Distortion of illusion of smell	Olfactory hallucinations	Olfactory agnosia
Parosmia or dysosmia: distortion of the perception of an odor. Cacosmia: perception of unpleasant odors (coprosmia, when it is the odor of feces).	Phantosmia: smelling odors that are not there.	Incapacity to discriminate odors, with preservation of perceptual aspects of primary smell.
e.g. temporal lobe epilepsy and smell recovery after neuronal damage of any kind.	e.g. temporal lobe epilepsy, psychiatric disorders and during smell recovery after viral etiology.	e.g. cortical lesions and Korsakoff syndrome.

## HOW TO EXAMINE AND INTERPRET FINDINGS? TESTING THE OLFACTORY NERVE

Obtaining the clinical history is essential, particularly in determining etiology (either in neurological diseases in general, or in uncovering the cause of olfactory dysfunction). However, attentive physicians who are determined to routinely examine olfaction will no doubt eventually be faced with the following situation: a subclinical or unreported olfactory dysfunction that was only detectable through physical examination. Hence, although neurological examination is seldom useful in pinpointing the exact site of the lesion (an exception in Neurology), meticulous testing of olfaction is rarely a futile effort, even among asymptomatic patients, considering that this may bring an otherwise unperceived smell deficit to the fore^15^.

Before starting the examination, the first step is always to ensure that both nostrils are clear and unobstructed. Check whether there is any kind of nasal congestion that could potentially alter adequate evaluation of olfactory nerve (ON) function (Table 3) and search for signs of trauma or other macroscopic alterations such as polyposis and deviated septum. These could steer the diagnosis towards a conductive cause for anosmia/hyposmia. Under standard inspiration, only a small amount of air will actually reach the olfactory mucosa inside the nose. Inhaling deeply and more rapidly helps to better direct the air towards the olfactory crypt and its receptors. Patients always need to be instructed in this regard and there is also a need to double check that they did not just “breathe on top” of the object that is being used in the test. Deep inspiration is critical in order to avoid false-negative findings^2^.

**Table 3.** Key points during the neurological examination.

Key points during neurological examinations
1) Check that nostrils are unobstructed and that the airflow is normal.
2) Present different odors to the patient, while closing one nostril at a time with your finger on the ipsilateral upper lateral cartilage. Ask for a quick and deep inhalation and then for identification of the odors.
3) Preferably, formally validated psychophysical tests should be performed (for example: UPSIT test).
4) Avoid using irritating substances like alcohol and ammonia.
5) Do not forget to examine all cranial nerves, with particular attention to the facial nerve (especially the gustatory portion), trigeminal nerve and optic nerve (fundoscopy should always be performed).
6) During the examination, search for signs of neurological or systemic diseases that may be commonly associated with olfactory loss (e.g. cleft lip in Kallmann syndrome, nystagmus due to phenytoin use, rigidity and tremor from Parkinson’s disease, muscular atrophy from amyotrophic lateral sclerosis or motor impersistence from Huntington’s disease).

UPSIT: University of Pennsylvania Smell Identification Test.

There is still ongoing discussion regarding the merits of testing each nostril individually. Some groups have argued against this, claiming that there is a large degree of mixing of the inhaled air in the nasopharynx, thus making separate nostril testing a pointless practice. Others have suggested that there is brief segregation of the stimulus in situations of quick inspiration, which adds value to individual nostril testing for detection of unilateral lesions^8^. Until this matter is settled, we advocate for the latter technique.

Most clinicians examine the ON through presenting patients with one or two odors (e.g., coffee or cinnamon) and asking them if they can accurately identify these odors. Should physicians choose this method of examination, they must keep in mind that this provides a very rudimentary assessment of smell function. It is comparable to testing visual acuity only by flashing a simple white light once in the direction of each eye, asking if the patient saw the light beam and believing that a thorough visual assessment was made. Although this method is certainly better than not testing at all, practical methods with much higher sensitivity are now available for evaluating the ON^16^.

Independently of which method is chosen, i.e. whether subjective and straightforward, like two-odor testing, or whether formal, like psychophysical olfactory tests, avoidance of irritant odorants should be an inflexible rule. Inhalation of particles such as alcohol (unfortunately, in our experience, often erroneously used for testing the ON) or ammonia stimulates trigeminal receptors, which both interferes with proper olfactory evaluation and generates a false sense of odor perception^8^.

### Olfactory testing

Modern psychophysical olfactory tests are the method most widely used in clinical practice. These are done by presenting several different types of odors to patients, who then, in turn, provides responses regarding smell identification, discrimination, threshold detection and memory. Odor identification tests (OITs) are the most reliable among these, since they are less cognitively demanding for patients^2^. 

Despite the immensely vast number of distinct smells in nature, these tests only use a small number of them. The theoretical basis for this approach comes from knowledge of the close proximity of olfactory pathways: lesions anywhere along the olfactory system pathways lead to impairment of the perception of multiple smells simultaneously, regardless of site. This precludes the need for testing with a thousand different odors while still giving the examiner an excellent general idea about patients’ ON function^16^.

OITs are valuable for providing an idea of the patient's olfactory spectrum, i.e. how many different types of smells they can accurately identify and discriminate between. This correlates remarkably well with the olfactory threshold, i.e. the amount of stimulus needed to create the sensation of smell. In other words, loss of ability to discriminate between different types of smells (microsmia) is also an indication that the patient has some degree of hyposmia (loss of function). So, this method serves to evaluate olfactory function quantitatively as well, and it has proved to be more reliable for detecting hyposmia than has subjective information gathered using one or two odors alone^17^.

UPSIT (University of Pennsylvania Smell Identification Test) is the most popular OIT^18^. It consists of 40 different odors and is administered in a multiple-choice format of questioning. There is also a smaller version of the test called BSIT (Brief Smell Identification Test)^19^, consisting of 12 smells. UPSIT is not only reliable (with adjustments for age and sex) but also simple, such that it can be self-administered, thus enabling home testing in extensive populations, in a trustworthy “do-it-yourself” system^2^. These characteristics make it an ideal method for testing patients in quarantine or who are socially distancing themselves, through online consultations: in short, in the situations that we have been facing during the SARS-CoV-2 pandemic.

Moreover, UPSIT helps in differentiating organic from functional olfactory loss, since it is mathematically expected that subjects should get at least ten questions right (25% of the test), out of the 40 questions presented (each question poses a choice between four options), even if they are entirely anosmic or are guessing at random. Patients with functional anosmia tend to give wrong answers to most or all of the questions, thus amounting to a score of fewer than ten correct answers. There are, however, some disadvantages to this method: the smells are exposed to both nostrils simultaneously, which theoretically makes it more challenging to detect unilateral loss. There is also a significant cultural bias concerning the odors selected^16^.

Another well-established test is Sniffin Sticks^20^, a nasal chemosensory performance test that uses pen-like odor dispensing devices. It includes 12, 16 or 32 items and, in its complete form, evaluates olfactory threshold, discrimination and identification. It correlates well with UPSIT, although it is more time-consuming and demands previously trained staff to apply it^17^.

Lastly, information gathered from the remainder of the neurological examination is always extremely valuable. Every patient who comes in with olfaction complaints needs to undergo careful examination of all of the remaining cranial nerves. 

Clinician should, at least, always test for deficits in the facial nerve (especially concerning gustatory function) and in the trigeminal and optic nerves. In particular, bilateral fundoscopic examination should be performed to search for optic atrophy or papilledema, which may point to an anterior frontal mass or tumor. It goes without saying that any other focal neurological signs or evidence of neurodegenerative diseases, parkinsonism or vitamin deficiencies (among others), should be explored and valued in the proper clinical context^2^.

## HOW TO LOCALIZE THE LESION?

Lesion localization has always been a staple of neurological practice. Precise localization not only narrows the differential diagnosis but also helps to determine which ancillary testing route will be pursued. Usually, in Neurology, clinicians will use information gathered in the physical examination to determine the lesion site and information from the history to determine etiology. 

In this regard, the clinical reasoning behind diagnosing olfactory disturbance is, once again, unconventional. The clinical history plays a greater role in localization than does the neurological examination, which more frequently helps in detection and proper documentation of the nature or severity of deficits.

Olfactory dysfunction can be caused by central or peripheral nervous system lesions. Similarly to auditory lesions, conductive olfactory loss is strictly caused by peripheral lesions, while sensorineural olfactory loss might be caused by either central alone or combined central and peripheral lesions (anywhere from the olfactory receptors in the nose to the olfactory cortex) (Table 4).

**Table 4.** Clinical pearls.

Clinical pearls for olfactory investigation
1) Lesions in any segment of the olfactory nerve usually affect the perception of more than a single stimulus, because of the proximity of the olfactory pathways.
2) The most reliable tests are those consisting of stimulus presentation that involves odor identification. These tests are therefore preferrable for clinical practice.
3) It is indispensable to test each nostril individually, in order to detect potential unilateral problems. It is essential to point out that patients with unilateral problems usually do not complain about olfactory loss.
4) Complaints of hyposmia or anosmia indicate a bilateral lesion.

### Peripheral olfactory impairment

The olfactory neuroepithelium consists of receptors and first-order neurons situated in the posterosuperior portion of both nasal cavities. This is the most common lesion site, with regard to both conductive and sensorineural olfactory loss^21^.

Conductive olfactory loss results from any cause that halts inspired airflow (and, with this, odor molecules) from reaching olfactory receptors. We have mentioned that mucosal integrity and an unobstructed pathway for air inhalation are both fundamental prerequisites for physiological olfaction. The most frequent causes of conductive olfactory loss include deviated septum, osteomeatal deformity owing to trauma, nasal polyps, nasal tumors, allergic rhinitis and chronic rhinosinusitis (CR). CR is fairly common, and 80% of the patients suffering from it have had some sort of olfactory dysfunction. Loss of function in this setting can result from conductive and/or sensorineural causes, respectively due to either nasal blockage or olfactory neuroepithelium damage^21^.

Peripheral damage causing sensorineural olfactory loss alone, without conductive abnormalities, is mainly secondary to viral or post-viral infections. URTI may cause olfactory dysfunction even in the absence of previous flu-like symptoms^21^, which means acute anosmia could be the sole symptom of an URTI. Examples of viral agents commonly involved include rhinoviruses, coronaviruses, parainfluenza and influenza viruses and Epstein-Barr virus^22^. It is worth pointing out that individuals presenting with viral URTI-induced olfactory dysfunction might also be predisposed to other cranial neuropathies^23^, which further proves the need for thorough cranial nerve examination in these patients. 

Sensorineural olfactory loss can result from other types of infectious etiologies (although much less common), such as bacterial pathogens. *Mycobacterium leprae* is a neglected, albeit common cause of anosmia in countries where this disease is prevalent. In a study conducted in Lucknow, India, it was concluded that all patients diagnosed with Hansen’s disease had some level of olfactory dysfunction^24^. So, in patients with the appropriate epidemiology, leprosy should always be in the differential diagnosis of anosmia. Fungal disease is also a relevant cause, especially among immunocompromised and diabetic patients (e.g., aspergillosis and mucormycosis)^25^.

Lastly, non-infectious diseases like tumors (e.g., small-cell carcinoma, adenoma or inverted papilloma) may also cause conductive or sensorineural olfactory loss. The classic example is esthesioneuroblastoma, a rare neuroepithelial tumor that arises from the olfactory neuroepithelium in the cribriform plate^26^.

### Central olfactory impairment

The first central nervous system structure in the olfactory pathways is the olfactory bulb. It is located in the anterior fossa, above the cribriform plate. Anterior fossa tumors and aneurysms of the anterior communicating artery or anterior cerebral artery are both central causes of anosmia or hyposmia resulting from olfactory bulb lesions^8^. The clinical presentations of these disorders are one of the better-known syndromes associated with olfactory dysfunction: ipsilateral anosmia and optic atrophy (from direct compression of the olfactory bulb and optic nerve), in association with contralateral papilledema (from increased intracranial pressure), i.e. the Foster-Kennedy syndrome^27^.

From the olfactory bulb, the olfactory pathways follow through olfactory tracts and are then processed in structures collectively referred to as the primary olfactory cortex, which is responsible for more complex olfactory processing. These are the anterior olfactory nucleus, piriform cortex, anterior cortical nucleus of the amygdala, periamygdaloid complex and rostral entorhinal cortex. Depending on the nature of the injury, dysfunction of these structures can result either in olfactory loss or high-order olfactory disturbances, such as olfactory hallucinations and olfactory agnosia. The leading causes of cortical olfactory loss are neurodegenerative, demyelinating, nutritional and metabolic disorders.

Recognizing early signs of olfactory loss secondary to degeneration in olfactory areas (including the olfactory bulb) in some neurodegenerative diseases can be very useful in clinical practice. For example, finding an olfactory deficit in a patient with mild cognitive impairment can predict progression to Alzheimer’s disease^8^^,^^28^. Moreover, the prevalence of olfactory dysfunction in Parkinson’s disease is so high that its absence should be a warning sign for the diagnosis^29^. The same principle can be applied to other degenerative diseases and may prove extremely helpful in the differential diagnosis. Progressive supranuclear palsy and corticobasal degeneration syndrome usually spare the sense of smell. In contrast, dementia with Lewy bodies, frontotemporal dementia, pure autonomic failure, Huntington’s disease and lateral amyotrophic sclerosis usually impair it^1^^,^^21^.

One must not, however, jump to hasty conclusions and assume that olfactory loss in an elderly patient necessarily equates to a neurodegenerative process. Although olfactory dysfunction is very prevalent in many neurodegenerative diseases, the opposite is not a rule: most hyposmic patients do not have life-threatening neurological conditions.

Demyelinating diseases (e.g., multiple sclerosis, neuromyelitis spectrum disorder and acute disseminated encephalomyelitis) have also been shown to be associated with olfactory dysfunction. In some exceptional cases, anosmia was the first manifestation of multiple sclerosis^21^^,^^30^. 

Metabolic/nutritional disorders, especially thiamine and vitamin A deficiencies, are also worth mentioning. These not only can cause olfactory loss but also, in patients with Korsakoff’s psychosis, can give rise to odor discrimination deficits and olfactory agnosia. This probably results from degeneration of the medial nuclei of the thalamus^2^.

Some conditions are known for their interesting and unique presentations, such as causing olfactory hallucinations without associated olfactory loss. Classical examples of this include migraine auras and temporal lobe epilepsy, with seizures presenting as positive olfactory phenomena, usually of unpleasant characteristics, like cacosmia (traditionally known as uncinate seizures)^31^.

### Combined central and peripheral olfactory impairment

A few conditions are responsible for olfactory deficits that combine central and peripheral mechanisms. Among these are head injury and several systemic diseases.

Head trauma can lead to an assortment of neurological conditions, such as concussion, cranial fractures, intracranial hematomas, damage to several cranial nerves, damage to arteries and intraparenchymal contusions. The olfactory system is no exception, and it can be affected in a multitude of ways. Lesions in the sinonasal area, lacerations of the olfactory nerve itself while passing through the cribriform plate, damage to the olfactory bulb through traction forces and hemorrhages of the orbitofrontal and anterior temporal lobe have all been reported as potential causes of hyposmia after traumatic head injuries. Particularly when presence of cerebrospinal fluid rhinorrhea is associated with the trauma, the risk of olfactory dysfunction seems to be higher. When the orbitofrontal cortex is concomitantly injured, associated symptoms such as dysexecutive and behavioral syndromes can occur^21^. Some patients with traumatic anosmia can also suffer from true traumatic ageusia, which, if detected on examination, could work as a clinical tip for the etiological diagnosis of olfactory loss, although the reason for this association remains unclear^8^.

Although anosmia secondary to systemic disease (endocrinological, renal, hepatic or rheumatological) is a well-described entity, it results from many possible mechanisms (central or peripheral) that are not yet fully understood. Rheumatological conditions such as Sjögren’s syndrome, Churg-Strauss syndrome, Behçet’s disease, systemic lupus erythematosus, scleroderma, giant-cell arteritis and granulomatosis with polyangiitis may cause anosmia due to vasculitis, a phenomenon that is believed to be particularly underdiagnosed^21^.

## ANOSMIA FROM COVID-19

Post-viral olfactory dysfunction is a recognized cause of acute olfactory loss, and it accounts for as much as 15-20% of all anosmia cases^1^. However, over the past year, the new coronavirus pandemic (SARS-CoV-2 disease, COVID-19) has led to an exponential rise in acute anosmia cases and may have significantly increased the incidence of this already common condition. It is estimated that anosmia can occur in up to 60% of COVID-19 cases^32^^,^^33^.

The clinical presentation of anosmia secondary to SARS-CoV-2 is not particularly different from that of many other previously known causes of viral/post-viral olfactory loss. But, since acute anosmia can likewise present without any respiratory/flu symptoms, it is now considered to be an important symptom of SARS-CoV-2 infection, which motivates testing and even social isolation measures among these patients^34^. Recovery of olfactory function in these cases seems to be good, and for most patients the outcome is complete spontaneous recovery^21^. 

Olfactory nerve infection may also form a route for the virus to spread to the central nervous system (trans-cribriform spread), thus possibly explaining cases of acute encephalitis following documented infection. The mechanism of central nervous system damage is believed to be related to immune-mediated effects induced by the virus^35^.

## GENERAL PROGNOSIS AND TREATMENT

The olfactory nerve has a well-known reputation for having great power of regeneration. This happens continuously, through basal stem cells located on the olfactory epithelium. 

The prognosis for recovery after olfactory dysfunction depends on the underlying cause of injury. Usually, acute injury to the olfactory nerve (e.g., URTI) that spares the basal stem cells is followed by spontaneous recovery of olfactory function in most individuals. However, this is not true for many other causes of olfactory loss such as neurodegenerative diseases, trauma and compressive lesions of the olfactory bulb, which can result in persistent olfactory dysfunction^8^.

There are plenty of types of treatment available for anosmia, which are beyond the scope of this review. Regarding treatment options for patients with acute olfactory loss related to SARS-CoV-2 infection, the treatment strategy classically used for other forms of viral anosmia, consisting of use of topical corticosteroids, is currently being avoided due to the risk of immunosuppression^14^^,^^34^.

During the pandemic, smell training techniques have gained ground as a simple and cheap type of “physical therapy”. This form of rehabilitation consists of olfactory stimulation using four different odors, in which the patient smells each one for at least ten seconds, two times a day over a four-month period. Studies have shown this technique to be beneficial in both post-viral and post-traumatic cases of olfactory loss, leading to improvement of wellbeing and depression among these patients^36^^,^^37^.

In conclusion, over the course of human history, tragedies such as disease and war have frequently been powerful driving forces for mankind. Scientific advances driven by calamity have transformed previously well-established points of view and have often illuminated subjects that were traditionally overlooked. Some such advances have even been responsible for entirely resetting society, thereby leaving long-lasting legacies. 

This new virus is no different. The 2020 pandemic brought with it a crystal-clear fact: olfaction should no longer be neglected, either in clinical practice or in research.

We hope that this misfortune will result in a new legacy for future generations of neurologists, through promotion of novel habits of careful physical examination and clinical reasoning, thus placing olfaction under the spotlight that it has always deserved. This is important not only in our day-to-day clinical practice, but also for further research, in order to enable new discoveries in the field of human olfaction. How many conquests could, literally, be under our noses?

## KEY POINTS

1. Information from the clinical history is better than data gathered from physical examinations for locating and diagnosing the cause of olfactory lesions.
2. Less than half of all patients with olfactory loss will complain of it. Therefore, active searching is mandatory.
3. Evaluating a small sample of odors gives us a good general idea of patients’ olfactory capacity.
4. Olfactory dysfunction can be of either central or peripheral origin; the latter is most common.
5. Smell training techniques are an effective treatment for post-viral olfactory loss.

## References

[B1] Campbell WW, Barohn RJ (2020). Dejong's the neurologic examination.

[B2] Hawkes CH, Doty RL (2009). The neurology of olfaction.

[B3] Walusinski O (2018). Joseph Hippolyte Cloquet 1787-1840 -physiology of smell: portrait of a pioneer. Clin Transl Neurosc.

[B4] Schultze M (1856). Über die endigungsweise des geruchsnerven und der epithelialgebilde der nasenschleimhaut. Ber Akad Wiss Berlin.

[B5] Buck L, Axel R (1991). A novel multigene family may encode odorant receptors: a molecular basis for odor recognition. Cell.

[B6] Fornazzari L, Mansur A, Schweizer TA, Fischer CE (2015). Proust and madeleine: Together in the thalamus?. Neurol Clin Pract.

[B7] Proust M (1992). In search of lost time, volume I, Swann’s way.

[B8] Ropper AH, Samuels MA, Klein J, Prasad S (2019). Adams and Victor's principles of neurology.

[B9] Doty RL (2009). The olfactory system and its disorders. Semin Neurol.

[B10] Parkinson J (2002). An essay on the shaking palsy. 1817. J Neuropsychiatry Clin Neurosci.

[B11] Domellöf ME, Lundin K-F, Edström M, Forsgren L (2017). Olfactory dysfunction and dementia in newly diagnosed patients with Parkinson's disease. Parkinsonism Relat Disord.

[B12] Fornazieri MA, Borges BBP, Bezerra TFP, Pinna FR, Voegels RL (2014). Main causes and diagnostic evaluation in patients with primary complaint of olfactory disturbances. Braz J Otorhinolaryngol.

[B13] Welge-Lüssen A, Wolfensberger M (2006). Olfactory disorders following upper respiratory tract infections. Adv Otorhinolaryngol.

[B14] Boesveldt S, Postma EM, Boak D, Welge-Luessen A, Schöpf V, Mainland JD (2017). Anosmia-a clinical review. Chem Senses.

[B15] Sanders RD, Gillig PM (2009). Cranial nerve I: olfaction. Psychiatry (Edgmont).

[B16] Doty RL (2015). Olfactory dysfunction and its measurement in the clinic. World J Otorhinolaryngol Head Neck Surg.

[B17] Doty RL (2015). Handbook of olfaction and gustation.

[B18] Doty RL, Shaman P, Kimmelman CP, Dann MS (1984). University of Pennsylvania smell identification test: a rapid quantitative olfactory function test for the clinic. Laryngoscope.

[B19] Menon C, Westervelt HJ, Jahn DR, Dressel JA, O'Bryant SE (2013). Normative performance on the Brief Smell Identification Test (BSIT) in a multi-ethnic bilingual cohort: a Project FRONTIER study. Clin Neuropsychol.

[B20] Hummel T, Sekinger B, Wolf SR, Pauli E, Kobal G (1997). 'Sniffin' sticks': olfactory performance assessed by the combined testing of odor identification, odor discrimination and olfactory threshold. Chem Senses.

[B21] Doty RL (2019). Smell and taste.

[B22] Suzuki M, Saito K, Min W-P, Vladau C, Toida K, Itoh H (2007). Identification of viruses in patients with postviral olfactory dysfunction. Laryngoscope.

[B23] Jitaroon K, Wangworawut Y, Ma Y, Patel ZM (2020). Evaluation of the incidence of other cranial neuropathies in patients with postviral olfactory loss. JAMA Otolaryngol Head Neck Surg.

[B24] Mishra A, Saito K, Barbash SE, Mishra N, Doty RL (2006). Olfactory dysfunction in leprosy. Laryngoscope.

[B25] Sharma P, Bhaisora KS, Pandey S, Srivastava AK, Pani KC, Sardhara J (2018). Aspergilloma mimicking olfactory groove meningioma. Asian J Neurosurg.

[B26] Dulguerov P, Allal AS, Calcaterra TC (2001). Esthesioneuroblastoma: a meta-analysis and review. Lancet Oncol.

[B27] Ciurea AV, Iencean SM, Rizea RE, Brehar FM (2012). Olfactory groove meningiomas: a retrospective study on 59 surgical cases. Neurosurg Rev.

[B28] Jung HJ, Shin I-S, Lee J-E (2019). Olfactory function in mild cognitive impairment and Alzheimer's disease: a meta-analysis. Laryngoscope.

[B29] Schapira AHV, Chaudhuri KR, Jenner P (2017). Non-motor features of Parkinson disease. Nat Rev Neurosci.

[B30] DeLuca GC, Joseph A, George J, Yates RL, Hamard M, Hofer M (2015). Olfactory pathology in central nervous system demyelinating diseases. Brain Pathol.

[B31] Coleman ER, Grosberg BM, Robbins MS (2011). Olfactory hallucinations in primary headache disorders: case series and literature review. Cephalalgia.

[B32] Mermelstein S (2020). Acute anosmia from COVID-19 infection. Pract Neurol.

[B33] Yan CH, Faraji F, Prajapati DP, Boone CE, DeConde AS (2020). Association of chemosensory dysfunction and Covid-19 in patients presenting with influenza-like symptoms. Int Forum Allergy Rhinol.

[B34] Gane SB, Kelly C, Hopkins C (2020). Isolated sudden onset anosmia in COVID-19 infection. A novel syndrome? Rhinology.

[B35] Panciani PP, Saraceno G, Zanin L, Renisi G, Signorini L, Battaglia L (2020). SARS-CoV-2: “Three-steps” infection model and CSF diagnostic implication. Brain Behav Immun.

[B36] Birte-Antina W, Llona C, Antje H, Thomas H (2018). Olfactory training with older people. Int J Geriatr Psychiatry.

[B37] Hummel T, Rissom K, Reden J, Hähner A, Weidenbecher M, Hüttenbrink K-B (2009). Effects of olfactory training in patients with olfactory loss. Laryngoscope.

